# The Roles of NDR Protein Kinases in Hippo Signalling

**DOI:** 10.3390/genes7050021

**Published:** 2016-05-18

**Authors:** Alexander Hergovich

**Affiliations:** Cancer Institute, University College London, Paul O’Gorman building, 72 Huntley Street, London WC1E 6BT, UK; a.hergovich@ucl.ac.uk; Tel.: +44-20-7679-0723; Fax: +44-20-7679-6817

**Keywords:** NDR, Nuclear Dbf2-related kinase, STK38, serine/threonine kinase 38, LATS, large tumour suppressor, Warts kinase, Hippo kinase signalling, Tricornered, Yes-associated protein

## Abstract

The Hippo tumour suppressor pathway has emerged as a critical regulator of tissue growth through controlling cellular processes such as cell proliferation, death, differentiation and stemness. Traditionally, the core cassette of the Hippo pathway includes the MST1/2 protein kinases, the LATS1/2 protein kinases, and the MOB1 scaffold signal transducer, which together regulate the transcriptional co-activator functions of the proto-oncoproteins YAP and TAZ through LATS1/2-mediated phosphorylation of YAP/TAZ. Recent research has identified additional kinases, such as NDR1/2 (also known as STK38/STK38L) and MAP4Ks, which should be considered as novel members of the Hippo core cassette. While these efforts helped to expand our understanding of Hippo core signalling, they also began to provide insights into the complexity and redundancy of the Hippo signalling network. Here, we focus on summarising our current knowledge of the regulation and functions of mammalian NDR kinases, discussing parallels between the NDR pathways in *Drosophila* and mammals. Initially, we provide a general overview of the cellular functions of NDR kinases in cell cycle progression, centrosome biology, apoptosis, autophagy, DNA damage signalling, immunology and neurobiology. Finally, we put particular emphasis on discussing NDR1/2 as YAP kinases downstream of MST1/2 and MOB1 signalling in Hippo signalling.

## 1. Introduction

**Figure 1:**
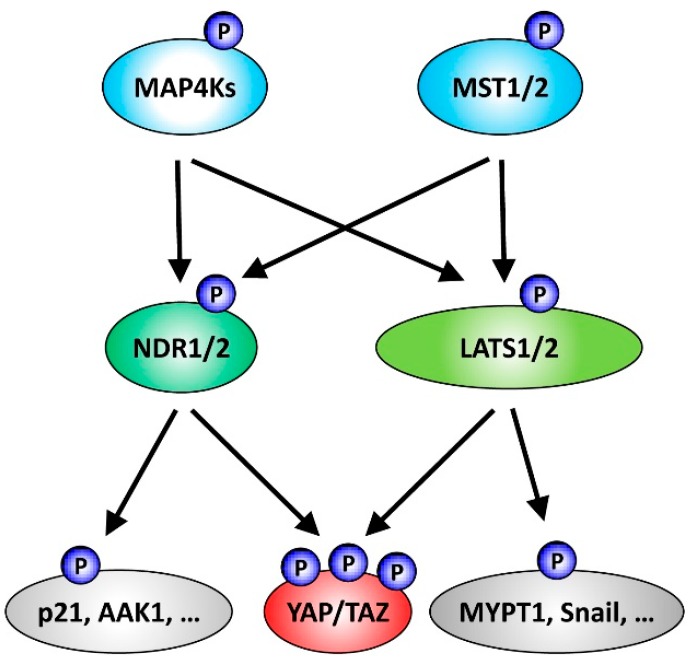
Summary of NDR/LATS kinase signalling functioning downstream of Ste20-like kinases and upstream of the Hippo effectors YAP/TAZ. Classically, the core cassette of the mammalian Hippo pathway includes the Ste20-like serine/threonine protein kinases MST1/2 and the AGC serine/threonine protein kinases LATS1/2. Active MST1/2-LATS1/2 signalling through LATS1/2-mediated phosphorylation of YAP/TAZ can inhibit the transcriptional co-activators YAP and TAZ, two major effectors of Hippo signalling. However, the list of kinases functioning as part of the Hippo core cassette has been expanded recently. In addition to MST1/2, members of the Ste20-like MAP4K kinase family can perform the activating phosphorylation of LATS1/2, consequently resulting in YAP/TAZ inhibition through LATS1/2-mediated phosphorylation. Moreover, the AGC serine/threonine NDR1/2 kinases can act as YAP kinases. Noteworthy, as outlined in more detail in the main text, MAP4K4 can also function upstream of NDR1/2, and NDR1/2 as well as LATS1/2 kinases phosphorylate additional substrates besides YAP.

More than a decade ago the Hippo pathway was discovered as a highly conserved signal transduction cascade that functions as a key co-ordinator of tissue growth control and homeostasis [1,2]. From a classical perspective, the core cassette of the mammalian Hippo pathway comprises the following signal transducers: the Ste20-like serine/threonine protein kinases MST1 and MST2 (also termed STK4 and STK3), the AGC serine/threonine protein kinases LATS1 and LATS2, and the SAV1 (also termed WW45) and MOB1 scaffold proteins [1,3]; with the transcriptional co-activators YAP and TAZ functioning as major effectors of Hippo signalling [4,5]. In its on-state, the Hippo pathway inhibits YAP/TAZ by MST1/2-SAV1-MOB1-LATS1/2 signalling through LATS1/2-mediated phosphorylation of YAP/TAZ on different serine residues, which can cause cytoplasmic retention and proteasome-mediated degradation of YAP/TAZ [6]. Once the Hippo pathway is turned off, MST1/2 do not activate LATS1/2, hence resulting in the release of YAP/TAZ from inhibitory phosphorylation events by LATS1/2, and subsequently in the nuclear accumulation of active YAP/TAZ. While this model of a linear signalling cascade served well for the initial studies of the Hippo pathway, recent research has uncovered additional kinases, such as the AGC serine/threonine NDR1/2 kinases (also known as STK38/STK38L) and members of the Ste20-like MAP4K family, as novel members of the core cassette of Hippo signalling (Figure 1). More specifically, together with MST1/2 various members of the MAP4K kinase family are responsible for the activating phosphorylation of LATS1/2 in order to inhibit YAP/TAZ through LATS1/2-mediated phosphorylation [7,8,9]. In addition, although LATS1/2 are the important YAP kinases in HEK293 cells [7], a recent study by Zhang *et al.* established NDR1/2 as additional YAP kinases using a combination of biochemical, cell biological and genetic approaches [10]. Cumulatively, these recent studies helped to expand our understanding of Hippo core signalling by providing insights into the complexity of the Hippo core cassette functioning upstream of YAP/TAZ (Figure 1). In this review, we will focus on summarising the connections of the NDR1/2 protein kinases with the Hippo pathway and provide an updated overview of cellular functions and substrates of NDR1/2.

## 2. Review

The first NDR serine/threonine kinase, termed Dbf2p, was discovered in budding yeast [11], followed by discoveries in *Neurospora crassa* [12] and human cells [13], while Tricornered (Trc), the fly counterpart of mammalian NDR1 and NDR2 kinases, was identified later [14]. Noteworthy, members of the NDR kinase family are so highly conserved that human NDR1 can rescue the loss-of-function phenotype of Trc-deficient flies [3,15]. Based on unique structural characteristics members of the NDR kinase family have been identified in diverse eukaryotes ranging from unicellular organisms such as yeast to complex multicellular organisms such as plants, animals and humans [3]. In this regard, one should note that NDR kinases are essential for the survival of many uni- and multicellular organisms. For example, NDR kinases are required for the viability of *Trypanosoma brucei*, the causative agent of human African trypanosomiasis [16], and genetic inactivation of Trc, the fly NDR kinase, results in larval lethality [14]. Furthermore, *Ndr1/2* double knockout mouse embryos display multiple phenotypes including defective somitogenesis and cardiac looping, resulting in a developmental delay from embryonic Day 8.5 (E8.5) onwards, followed by embryonic lethality around E10 [17]. Therefore, it is important to understand the molecular and cellular functions of NDR kinases in different species.

### 2.1. The Regulation of Mammalian NDR1/2 Kinases

Initially, little was known about the *in vivo* functions of mammalian NDR1/2 kinases, but extensive biochemical studies have been carried out to understand the molecular regulation of NDR1/2 kinases, which up to recently [18,19] had to serve as a model for the regulation of LATS1/2 kinases [20]. In summary, it was discovered that MST1, MST2 and MST3 can phosphorylate NDR1/2 on Thr444/Thr442 in their hydrophobic motifs, while the binding of MOB1 to NDR1/2 through the highly conserved NTR domain, located proximal to the catalytic domain of NDR1/2, is required to support the auto-phosphorylation of NDR1/2 on Ser281/Ser282 in their T-loop (summarised in [3,20,21]). Noteworthy, the experimental activation of NDR1/2 can be achieved through different routes involving the inhibition of protein phosphatase 2A (PP2A) [22], the mutation of an autoinhibitory segment juxtaposed to the T-loop phosphorylation site [23], membrane targeting of NDR1/2 [24], and through modifications of the hydrophobic motif [25,26]. Therefore, NDR1/2 can be regulated through different molecular mechanisms involving changes in their subcellular distribution and phosphorylation status (modified by upstream activators such as MST1/2/3 and inhibitors such as PP2A). Nevertheless, NDR1/2 kinases are possibly also regulated by post-translational modifications other than phosphorylation, including ISGylation of NDR1 [27] and ubiquitination and acetylation of NDR1/2 as proposed by the Cell Signaling database (www.phosphosite.org). However, the functional significance of these additional post-translational modifications is yet to be established.

### 2.2. Biological Functions of Mammalian NDR1/2 Kinases

#### 2.2.1. Roles of NDR1/2 in Cell Cycle Progression and Cell Cycle Associated Processes

Experiments using tissue culture cells indicate that NDR1/2 can play diverse roles in processes associated with the mammalian cell cycle. Through the regulation of c-myc and p21/Cip1 protein levels, NDR1/2 have been linked to the regulation of G1/S cell cycle progression [28,29,30,31], with p21/Cip1 as NDR1/2 substrate (Table 1 and Table 2). NDR1/2-mediated G1/S cell cycle progression is supported by cyclin D1 [32] and can oppose a TGFβ-mediated cell cycle arrest [33]. NDR1 can further play a role in mitosis [34,35] through NDR1/2-mediated phosphorylation of heterochromatin protein 1α (HP1α, also known as CBX5) [36] (Table 1 and Table 2) and NDR1 functioning downstream of PLK1 in mitotic cells [37]. In addition, the cell cycle dependent localisation of NDR1/2 to centrosomes [38] can support centrosome duplication in S-phase [26,38,39]. Moreover, NDR2-mediated phosphorylation of Rabin8 supports primary cilia formation [40] (Table 1 and Table 2), which supports a possible role of defective NDR2 signalling in ciliopathy [41,42], since primary cilia, as cell cycle regulated antenna-like sensory structures with a centrosome base, play important roles in signal transduction [43,44] and disease-associated processes [45,46].

#### 2.2.2. Roles of NDR1/2 in Apoptosis, Stress Signalling and Autophagy

Current reports suggest that NDR1/2 can function as pro-apoptotic kinases downstream of Ste20-like kinases [47,48,49,50,51,52], with MICAL-1 interfering with MST1-mediated phosphorylation (activation) of NDR1/2 in apoptotic cells [50,51]. Furthermore, NDR1/2 contribute to stress signalling [49,53] and play a role in autophagy as a major stress response [48]. More specifically, NDR1 and Trc are required for early autophagosome formation in human cells and fly larvae, respectively [48], a process which most likely involves Beclin1 and ULK1 as major regulators of autophagy [54]. In addition, NDR kinases can mediate mitochondrial quality control in flies and human cells [55], suggesting that NDR1/2 may sustain selective autophagic processes such as mitophagy. However, the downstream effectors of NDR1/2 in apoptosis and autophagy signalling are yet to be defined.

#### 2.2.3. Roles of NDR1/2 in DNA Damage Signalling

By competing with MOB1 for binding to NDR1/2, MOB2 can interfere with NDR1/2 activation [56]. Since MOB2 can function as a DNA damage response (DDR) factor [57], this could suggest that NDR1/2 may also function in the DDR, though this does not seem to be the case, since the DDR role of MOB2 appears to be independent of NDR1/2 signalling [57]. Nevertheless, we believe that only further investigations can completely rule out that NDR1/2 signalling is not linked to the DDR through MOB2, since recent reports already suggest an involvement of NDR1/2 in the DDR. Specifically, NDR1 can interact with XPA (Xeroderma pigmentosum A protein), thereby possibly playing a role in the nucleotide excision repair, a specific type of DNA damage repair [58]. NDR1-depleted HeLa cells displayed increased sensitivity to ionizing radiation [59], which potentially is linked to NDR1 as a HSP90 client [59,60]. Moreover, NDR1 appears to be involved in the activation of the DNA damage induced G2/M cell cycle checkpoint by phosphorylating the CDC25A phosphatase on Ser76 [61] (Table 1 and Table 2). However, considering that the phosphorylation of CDC25A on Ser76 is also mediated by CHK1 to promote CDC25A degradation by the SCF^β^^TrCP^ E3 ligase [62], the regulation of the G2/M checkpoint by NDR1/2 deserves further investigation. In this regard, it is already well-established that PLK1 is required for CDC25 activation to initiate the recovery from the G2/M checkpoint [63,64], a process which possibly is linked to the regulation of NDR1/2 by PLK1 [37] and the connection between NDR1/2 and CDC25A [61].

#### 2.2.4. Roles of NDR1/2 in Immunology

Initially, it was observed that the HIV-1 virus associates with NDR1/2 [65], however, the biological significance of this interaction remained unknown. Recent studies of mouse models [52,66,67] have started to shed light on the involvement of NDR1/2 in immunological processes, hence suggesting that the association of HIV-1 with NDR1/2 might play a role in the modulation of the immune system by the virus, although this hypothesis is yet to be addressed experimentally. *Ndr1* knockout mice are prone to develop T cell lymphomas, possibly due to defective apoptotic signalling in T lymphocytes [52]. An independent study of *Ndr1* knockout mice further found that NDR1 contributes to the innate immune response of macrophages [66]. In addition, using mice with T cell specific double knockout of *Ndr1/2*, Tang *et al.* found that NDR1/2 play important roles in thymocyte egress and migration [67].

#### 2.2.5. Roles of NDR1/2 in Neurobiology

Members of the NDR family play key roles in the morphogenesis of polarised structures in yeast, worms and flies [3], suggesting that NDR kinases may contribute to the biology of highly polarised mammalian cells such as neurons. Initial discoveries of NDR1/2 (Trc) signalling in neuronal development and maintenance were made in flies [68]. It was found that Trc, the fly NDR kinase, is required for dendritic branching and tiling in *Drosophila* sensory neurons [69], functioning downstream of the Hippo kinase [70] and TORC2 (target of rapamycin complex 2) in dendritic tiling [71]. Subsequently, by employing chemical genetics, Ultanir *et al.* could demonstrate that NDR1/2 are also important for dendrite growth and spine development in mammals by phosphorylating AAK1 and Rabin8 [72] (Table 1 and Table 2). By phosphorylating the polarity protein Par3 (Partitioning defective 3) NDR1/2 can further regulate neuronal polarity [73] (Table 1 and Table 2). Moreover, animal studies found that NDR2 plays a role in integrin-dependent neurite outgrowth in mice [74] and that NDR1/2 may act in rat dendrites [75].

### 2.3. The Connections between NDR Kinases and Hippo Core Signalling

As outlined above, NDR kinases have diverse biological functions, some of which are potentially interlinked with the Hippo pathway. In the following subsections, we provide an overview on how and to which degree fly and mammalian NDR kinases are linked to the core cassette of the Hippo pathway and hence why NDR kinases should be considered as novel core components of Hippo signalling.

#### 2.3.1. NDR Kinases as YAP Regulators

In *Drosophila*, Trc is the counterpart of mammalian NDR1/2 and the Warts kinase corresponds to mammalian LATS1/2, while Yorkie (Yki) and Mats are the orthologues of mammalian YAP/TAZ and MOB1, respectively. Since Trc/Warts double mutant cells displayed additive phenotypes combining the overgrowth phenotype (normally associated with Warts loss-of-function) and the wing hair phenotype (normally associated with Trc mutant), it was proposed that the Trc and Warts pathways function in parallel in fly wing cells [80]. Nevertheless, in spite of these clear differences between the Trc and Warts pathways in fly tissues, there are also connections in some contexts. One connection is that Hippo, the fly MST1/2 kinase, acts upstream of Trc and Warts in fly neurons [70]. A second connection is that Trc and Warts mutant cells display elevated levels of F-actin [80,81], a phenotype also observed upon Yki overexpression or Hippo loss-of-function [80]. A third connection is that Trc and Warts mutant cells show upregulated DE-Cadherin expression, which was also observed in Mats mutant cells [80]. Although Yki overexpressing cells displayed multiple hair as well as overgrowth phenotypes as observed in Trc and Warts mutant cells [80], Trc mutant cells did not display altered expression of well-established readouts for Yki activity such as *diap1*, *cyclin E* and *expanded* [80]. Thus, Fang *et al.* concluded that Trc does not regulate Yki in the fly wing [80], although whether Trc could phosphorylate Yki was not tested.

In mammalian cells, unlike LATS1/2 overexpression, the overexpression of NDR1/2 did not result in a phospho-shift of YAP [82]. Thus, it was believed that NDR1/2 do not function as YAP kinases in mammals [82], but again a possible connection between NDR1/2 and YAP was not further tested biochemically or genetically in this report. Subsequently, Zhang *et al.* addressed this possible connection experimentally, resulting in the discovery that mammalian NDR1/2 can function as YAP kinases [10]. They found that NDR1/2 directly phosphorylate YAP on Ser61, Ser109, Ser127 and Ser164 (Table 1 and Table 2); all four sites being also phosphorylated by LATS1/2 [76,82], hence establishing NDR1/2 as novel *bona fide* upstream kinases of YAP *in vitro*. Significantly, NDR1/2-mediated phosphorylation of YAP on Ser127 resulted in the inactivation of YAP by cytoplasmic retention [10], as reported for the LATS1/2-mediated phosphorylation of YAP on Ser127 [76,77], thereby establishing on the cellular level that NDR1/2 can function upstream of YAP. Even more importantly, the genetic ablation of *Ndr1* and *Ndr2* in the murine intestinal epithelium resulted in elevated total YAP protein levels accompanied by decreased Ser127 phosphorylation without any alterations in LATS1/2 phosphorylation (either on the T-loop or hydrophobic motifs) and total LATS1/2 protein levels [10]. Significantly, the increased YAP levels in NDR1/2-deficient tissue translated into increased YAP activity *in vivo* as judged by nuclear accumulation of YAP and elevated YAP target gene expression [10]. Moreover, the concomitant ablation of *Yap* in *Ndr1/2* double knockout tissue resulted in decreased tumour incidences upon chemically-induced colon carcinogenesis [10], suggesting that NDR1/2 are required to restrict the oncogenic potential of YAP in the intestinal epithelium. Taken together, this study [10] established NDR1/2 as novel YAP kinases.

Considering that the regulation of YAP by LATS1/2 has also been established on the biochemical, cellular and organismal levels [76,77,82,83], it is now imperative to establish in which context and settings LATS1/2 and/or NDR1/2 function as YAP kinases. Studies employing mouse genetics have so far shown that NDR1/2 are YAP kinases in the intestine [10] and that LATS1/2 can act as YAP kinases in the liver [83], hence many more studies using mouse models are warranted to investigate the potentially tissue specific roles of NDR1/2 and LATS1/2, both during normal growth and tissue homeostasis, and in tumourigenesis. In this context, it will be very interesting to understand how NDR1/2 can regulate total YAP protein levels without phosphorylating YAP on its phospho-degron motif (Ser381/397 in human cells and Ser366 in mice). Equally important will be to expand to studies of mammalian *Lats1/2* and *Ndr1/2* knockout cell lines. Currently we know that Ser127 phosphorylation of YAP is abolished, while total YAP phosphorylation still occurs to a significant extent in *Lats1/2* double knockout mouse embryonic fibroblasts [84]. In human *Lats1/2* double knockout HEK293 cells Ser127 and total YAP phosphorylation is dramatically diminished in different tissue culture conditions [7], which lead to the suggestion that LATS1/2 are the major YAP kinases [6]. However, we would like to stress that much more work in different cellular and organismal systems will be required to conclusively draw conclusions about the relative contributions of LATS1/2- and/or NDR1/2-mediated phosphorylation of YAP. In this regard, it will also be important to define any possible link between NDR1/2 and the regulation of TAZ, since NDR2, like LATS2 and MOB1B, is a target of the oncogenic microRNA-135b, which can play a role in TAZ deregulation and lung cancer progression [85]. In other words, decreased NDR2 levels have been associated with increased TAZ activity [85], but whether NDR1/2 act as TAZ kinases is yet to be determined. Taken together, NDR1/2-mediated phosphorylation of YAP and TAZ may play roles in different cell biological processes that are regulated by NDR1/2. However, except for the colon carcinogenesis model discussed above [10], the dependencies of NDR1/2 loss-of-function phenotypes on YAP/TAZ are yet to be investigated experimentally.

#### 2.3.2. NDR Kinases Functioning Downstream of Hippo and Hippo-Like Kinases

As already mentioned, Hippo, the fly MST1/2 kinase, can act upstream of Trc and Warts in fly neurons [70]. Specifically, Hippo phosphorylates Trc and Warts on their hydrophobic motif regulatory sites [70]. Very similarly, in mammals MST1/2 can act as regulators of NDR1/2 kinases in centrosome [39], apoptotic [47,50,51] and immune signalling [67] by functioning as hydrophobic motif kinases of NDR1/2 [20]. MST1/2 also phosphorylate LATS1/2 on their respective hydrophobic motif regulatory sites [19], which is important for LATS1/2 activation [19,86,87]. In this regard, it is noteworthy that mouse genetics have yet to confirm that MST1/2 can act as upstream kinases of LATS1/2 *in vivo*, while current evidence already suggest that MST1/2 function as upstream kinases of NDR1/2 *in vivo* in murine T cells [67]. Moreover, ablation of *Ndr1/2* results in intestinal epithelium hyperplasia [10] as observed in *Mst1/2* knockout mice [88,89], and *Ndr1/2* double null mouse embryos display multiple phenotypes [17] that are very similar to *Mst1/2* double null mouse embryos [90,91,92]. Thus, it appears that NDR1/2 can function as major effectors of MST1/2 signalling in different biological settings. Nevertheless, one should note that liver overgrowth and tumourigenesis were observed in *Lats1/2* and *Mst1/2* knockout mice [83,91,92,93], suggesting that LATS1/2 can also act as major effectors downstream of MST1/2 signalling *in vivo*.

Intriguingly, NDR1/2 and LATS1/2 are also regulated by other members of the Ste20-like kinase family besides MST1/2. First, MST3 can act as a hydrophobic motif kinase of NDR1/2 [29,94], while MST3 does not seem to function upstream of LATS1/2 [7]. Second, MAP4Ks can function as upstream hydrophobic motif kinases of NDR1/2 [49] and LATS1/2 or Warts in human cells and *Drosophila*, respectively [7,8,9]. More specifically, MAP4K4 functions as a hydrophobic kinase of NDR1/2 in osmotic stress signalling [49] and MAP4K4 was identified as a hydrophobic motif kinase of LATS1/2 in an unbiased kinome screen [7]. However, it turned out that *Map4k4* deletion in HEK293 cells does not affect YAP phosphorylation [7], hence Guan and colleagues investigated other members of the MAP4Ks family, revealing that several members of the MAP4Ks family can function upstream of LATS1/2 [7]. Nevertheless, in confluence tissue culture conditions residual Ser127 phosphorylation of YAP is still detectable in MM-8KO HEK293 cells (cells with knockouts of the *Map4k1/2/3/4/6/7* and *Mst1/2* genes), suggesting that another upstream kinase of LATS1/2 is yet to be identified [7]. In this regard, one should also consider another possibility, namely that a kinase other than LATS1/2 is phosphorylating YAP on Ser127 in this setting, with NDR1/2 being promising candidates to fulfil this role (see Section 2.3.1 above).

#### 2.3.3. NDR Kinases Regulated by the MOB1 Scaffold

In flies, both Trc and Warts are regulated by Hippo, a key member of the Hippo core cassette (see Section 2.3.2 above). Intriguingly, Trc and Warts have been physically and genetically associated with a second key member of the Hippo core cassette. Mats (also known as dMOB1) can interact with Trc and Warts, and is required for Trc and Warts functions in flies [81,95]. Trc, Warts and Mats play important roles in fly wing development with Mats mutant cells displaying phenotypes that were typical of Trc deficiency (*i.e.*, multiple wing hair cells) and of Warts loss-of-function (*i.e.*, tissue overgrowth and increased Yki target expression), strongly suggesting that in flies Mats is required for Trc as well as Warts signalling [81].

In mammalian cells, MOB1, encoded by the distinct *Mob1a* and *Mob1b* genes [21], can interact with NDR1/2 and LATS1/2 through a highly conserved NTR domain located proximal to the catalytic domain [19,23,24,39,56,87,96,97,98,99,100], with complex formation between MOB1 and the NDR1/2 or LATS1/2 kinases being essential for kinase activation through the stimulation of auto-phosphorylation [19,23,24,39,56,87,101,102]. Significantly, current evidence further suggests that MOB1 is a signal transducer scaffold bridging MST1/2-NDR1/2 [39,47] and MST1/2-LATS1/2 signalling [6,18], although an alternative model has been published recently [103] that challenges whether Hippo (MST1/2) and Warts (LATS1/2) complex formation is essential for Hippo signalling in fly tissues. More specifically, using a conformation sensor Vrabioiu *et al.* found that Warts/Mats complex formation is essential for Warts activation, while the stable interaction with Hippo appears to be dispensable. In this context, one should further note that the MST1/2-mediated phosphorylation of MOB1 on Thr12 and Thr35 is required to support the formation of NDR/MOB1 and LATS/MOB1 complexes [104]. In addition, by using membrane-targeted versions of MOB1 it was observed that the recruitment of NDR1 and LATS1 to membrane fractions by MOB1 is sufficient to significantly elevate NDR1 and LATS1 kinase activities [24,87], an effect also observed in fly tissues [105,106].

Recent studies of *Mob1a/b* knockout mice have revealed that MOB1 loss-of-function results in the most severe deregulation of the Hippo pathway *in vivo* [107,108], hence suggesting that the MOB1 scaffold represents a central hub of Hippo signalling. However, although MOB1 has clearly been linked to the NDR/Trc and LATS/Warts pathways in mammalian cells and fly tissues (see paragraphs above), the status of NDR1/2 signalling is yet to be studied in *Mob1a/1b* knockout mice. Therefore, future investigations are warranted to dissect the biological importance of MOB1 interactions with NDR1/2 and LATS1/2 in Hippo signalling.

#### 2.3.4. Comparison of NDR1/2 and LATS1/2 Regulatory Mechanisms in Hippo Signalling

As summarised in Section 2.1, the NDR1/2 kinases are regulated by different molecular mechanisms. Significantly, the regulatory mechanisms of LATS1/2 share striking similarities with the regulation of NDR1/2. Specifically, the experimental activation of NDR1/2 and LATS1/2 can be achieved through PP2A inhibition [19,22,25,87], membrane targeting [24,87], and modifications of the hydrophobic motif [19,25,26]. In addition, different lines of evidence clearly indicate that the NDR and LATS pathways can be regulated by the same members of the Hippo core cassette. First, both pathways are regulated by MST1/2 (Hippo) in mammalian cells and fly tissue (Section 2.3.2). Second, the NDR and LATS pathways are dependent on the support of MOB1 (Mats) (Section 2.3.3). Moreover, NDR1/2 and LATS1/2 can function as YAP kinases (Section 2.3.1). Therefore, it is very tempting to draw the conclusion that NDR1/2, like LATS1/2, should be considered as members of the Hippo core cassette.

As already summarised above, the NDR and LATS pathways are regulated through molecular mechanisms involving different members of the Hippo core cassette, which influence the phosphorylation status of NDR1/2 and LATS1/2. Interestingly, these similarities can be further extended to additional regulators of NDR1/2 and LATS1/2. For example, the RASSF1A (RAS association domain family 1A) tumour suppressor can trigger apoptotic signalling through MST1/2-mediated activation of LATS1/2 [109,110,111,112] and NDR1/2 [47]. Moreover, RASSF6 can inhibit the MST2-mediated activation of NDR1 and LATS2 [113], and both NDR1/2 and LATS1/2 seem to be HSP90 clients [59,60,114]. Thus, much is yet to be understood regarding how these diverse molecular players differentiate between the NDR and LATS signalling branches. For instance, besides understanding how additional regulators such as RASSF1A, RASSF6 or HSP90 play their specific roles, we have yet to uncover how MOB1, as a central hub of the Hippo pathway, may differentially interact with NDR1/2 and LATS1/2 and thereby potentially play diverse tumour suppressive roles. In this regard, we will need to define the molecular characteristics and cellular importance of the MOB1 interactions with MST1/2, LATS1/2 and NDR1/2 in Hippo signalling. Recent structural work on MST2 and LATS1 protein fragments in complex with MOB1 [18] together with the structure of the yeast NDR/LATS kinase Cbk1p [115] has started to shed light into the structural biology of the Hippo core cassette. Nevertheless, we have yet to complete our partial structural and molecular understanding of these important processes. In particular the complex formation of MOB1 with NDR1/2 is yet to be understood in the context of Hippo signalling.

## 3. Conclusions and Future Outlook

Taken together, based on the current evidence, one should consider the mammalian NDR1/2 kinases as novel members of the Hippo core cassette (at least in the context of intestinal epithelial cells). A key remaining question is to investigate in which other cell types and contexts NDR1/2 may function as YAP kinases downstream of Hippo core components such as MST1/2 and MOB1. In this regard, it might also be worth assessing whether Trc functions as a Yki regulator in specific fly tissues. Another interesting question will be to understand whether the regulation of the Hippo core cassette involving NDR1/2 is conserved throughout all eukaryotes. For example, like human NDR1/2 [22,25,116], Trc requires auto- and hydrophobic motif-phosphorylation for functionality [15]. In the filamentous fungus *Neurospora crassa*, the NDR kinase COT1 is also regulated by interacting with a MOB co-activator that supports T-loop auto-phosphorylation and by hydrophobic motif phosphorylation mediated the Ste20-like kinase POD6 [117]. However, the molecular regulation of the Hippo core cassette is yet to be understood in many other model organisms.

An additional key question should be the investigation of the potential regulatory crosstalk between the NDR and LATS pathways. Intriguingly, fly genetics could show that heterozygosity of a Warts mutant can enhance the Trc dominant negative phenotype in fly wings [81]. Moreover, it has been reported that, in yeast, the counterparts of the NDR and LATS pathways can crosstalk [118]. Thus, it will be interesting to explore any coordination and potential redundancy of NDR1/2 and LATS1/2 signalling in the Hippo and other important pathways. In this regard, another question is the regulation of transcriptional programmes by NDR1/2. Does NDR1/2 signalling regulate transcriptional regulators, besides YAP? TAZ should be investigated as a possible NDR1/2 substrate (see Section 2.3.1) as well as other transcriptional regulators, since NDR1/2 can interact with FIZ1 (Flt3 interacting zinc finger protein-1) in the nucleus [119]. Therefore, in addition to regulating cellular processes through the phosphorylation of substrates functioning in the cell cycle and other molecular machineries (Table 1 and Table 2), the regulation of transcriptional programmes by NDR1/2 signalling may have broader implications than currently appreciated. Consequently, it will be imperative to discriminate between subcellular functions that are dependent and/or independent of NDR1/2 kinase activities.

## Figures and Tables

**Table 1:** Summary of targeting motifs of NDR1/2 substrates in mammalian cells.

Targeting motif	Target site
*H*V*R*GD**pS**	YAP1 (human) on Ser61 [10]
*H*S*R*QA**pS**	YAP1 (human) on Ser109 [10]
*H*V*R*A*H***pS**	YAP1 (human) on Ser127 [10]
*H*L*R*QS**pS**	YAP1 (human) on Ser164 [10]
*HRR*IL**pS**	AAK1 (human) on Ser635 [72] #
*H*T*R*N*K***pS**	Rabin8 (mouse) on Ser240 [72] #
*H*T*R*N*K***pS**	Rabin8 (human) on Ser272 [40]
*KRR*QT**pS**	p21/CIP1 (human) on Ser146 [29]
LQ*R*MG**pS**	CDC25A (human) on Ser76 [61]
SPG*R*F**pS**	Par3 (mouse) on Ser383 [73]
QSG*RH***pS**	Par3 (human) on Ser1196 [73]
*RK*SNF**pS**	HP1α (human) on Ser95 [36] ##
*H*X*R*XX**pS/T**	proposed consensus motif [20]

Basic (positively charged) residues are highlighted in italic and present in each motif, although the HXRXXS/T signature is not present in all substrates; # PI4KB (pSer277), Panx2 (pSer514), and Rab11fip5 (pSer307) sequences are not shown, as they are not confirmed as direct substrates of NDR1/2, although all three substrates match the HXRXXS/T motif [72]; ## HP1α (also termed CBX5) is most likely phosphorylated by NDR1 on additional sites [36].

**Table 2:** Summary of direct downstream events/substrates of NDR1/2 kinases.

Substrate	Role of phosphorylation
YAP on Ser61	Not yet determined
YAP on Ser109	Not yet determined
YAP on Ser127	Facilitates cytoplasmic retention [10,76,77,78]
YAP on Ser164	Not yet determined
AAK1 on Ser635	Dendrite and spine development in neurons [72]
Rabin8 on Ser240	Dendrite and spine development in neurons [72]
Rabin8 on Ser272	Primary cilia formation [40]
p21/CIP1 on Ser146	Regulates p21/CIP1 protein stability [29]
CDC25A on Ser76	Regulates CDC25A protein stability [61] #
Par3 on Ser383	Regulates neuronal polarity [73]
HP1α on Ser95	Regulates mitotic progression [36]

# It remains to be clarified whether NDR1-mediated phosphorylation of CDC25A on Ser76 primes the subsequent phosphorylation of CDC25A on Ser79, Ser82 and Ser88 by NEK11, as already reported for CHK1-NEK11 signalling [62,79].

## Abbreviations

The following abbreviations are used in this manuscript:

AGC	protein kinase A (PKA)/PKG/PKC-like
Cip1	cyclin-dependent kinase interacting protein 1
HP1	Heterochromatin protein 1
HSP90	Heat-shock protein 90
LATS	large tumour suppressor protein kinase
MAP4K	Mitogen-activated protein kinase kinase kinase kinase
MICAL-1	molecule interacting with CasL-1
MOB1	Mps one binder 1
MST	Mammalian serine/threonine sterile 20 (Ste20)-like kinase
NDR	nuclear Dbf2-related protein kinase
Par3	Partitioning defective 3
PLK1	Polo-like kinase 1
PP2A	protein phosphatase 2A
RASSF	RAS association domain family
STK	serine/threonine protein kinase
TAZ	transcriptional coactivator with PDZ binding motif
TGF	transforming growth factor
Trc	Tricornered
ULK1	Unc-51 like protein kinase 1
YAP	Yes-associated protein
Yki	Yorkie
